# The involvement of J-protein AtDjC17 in root development in *Arabidopsis*

**DOI:** 10.3389/fpls.2014.00532

**Published:** 2014-10-08

**Authors:** Carloalberto Petti, Meera Nair, Seth DeBolt

**Affiliations:** Department of Horticulture, University of KentuckyLexington, KY, USA

**Keywords:** root hair, root patterning, development, heat shock protein, J-family, J-proteins

## Abstract

In a screen for root hair morphogenesis mutants in *Arabidopsis thaliana* L. we identified a T-DNA insertion within a type III J-protein AtDjC17 caused altered root hair development and reduced hair length. Root hairs were observed to develop from trichoblast and atrichoblast cell files in both *Atdjc17* and *35S::AtDJC17.* Localization of gene expression in the root using transgenic plants expressing pro*AtDjC17::GUS* revealed constitutive expression in stele cells. No *AtDJC17* expression was observed in epidermal, endodermal, or cortical layers. To explore the contrast between gene expression in the stele and epidermal phenotype, hand cut transverse sections of *Atdjc17* roots were examined showing that the endodermal and cortical cell layers displayed increased anticlinal cell divisions. Aberrant cortical cell division in *Atdjc17* is proposed as causal in ectopic root hair formation via the positional cue requirement that exists between cortical and epidermal cell in hair cell fate determination. Results indicate a requirement for *AtDJC17* in position-dependent cell fate determination and illustrate an intriguing requirement for molecular co-chaperone activity during root development.

## INTRODUCTION

During *Arabidopsis thaliana* L. Heyne root development, the distribution of hair cells has been extensively studied as a position-dependent developmental program. Here, the alternating emergence of trichoblast cells or root hair (H) versus. atrichoblast cells or non-root hairs (N) relies on positional information, whereby epidermal cells in direct contact with anticlinal cell walls of two underlying cortical cells acquire H cell fate and all others become N cells (Dolan et al., 1994; Dolan, 2006). This position-dependent cell fate determination at the root epidermis is reliant on a complex regulatory pathway involving genetic cell fate determinants and mobile transcriptional regulators (Kwak et al., 2005; Kwak and Schiefelbein, 2007). Here, SCRAMBLED (SCM), a leucine-rich receptor-like kinase (LRR-RLK) relays positional information underlying this pattern (Lee and Schiefelbein, 2002; Dolan, 2006; Kwak and Schiefelbein, 2008). Signaling through SCM establishes repression of the R2R3-MYB transcription factor WEREWOLF (WER) in H cells. As a result, the repression of WER in H cells causes increased levels of WER in N cells and in turn the levels of a regulatory complex consisting of transcriptional mediators WER-GLABRA3 (GL3)/ENHANCER OF GLABRA3 (EGL3)-TRANSPARENT TESTA GLABRA1 (TTG1). The combined effect of these factors causes activation of direct targets including the N-cell determinant and homeodomain-leucine-zipper transcription factor GLABRA2 (GL2; Kwak and Schiefelbein, 2008).

The complexity of cell fate determination in the epidermal layers is clearly evidenced by the large number of transcription regulators involved (Guimil and Dunand, 2006; Bruex et al., 2012), as well as by an increasingly large number of additional genes, e.g., auxin responsive, *AXR2, AXR3* (Nagpal et al., 2000; Knox et al., 2003), *KEU,* which encodes the yeast *Sec1* homolog, a key regulator of vesicle trafficking (Assaad et al., 2001), *ROP2* a small Rho-plant GTPase implicated in cytoskeleton organization and cell polarity (Jones et al., 2002, 2006) and the ethylene oxide genes *ETO1* and *ETO2* involved in ethylene synthesis (Cao et al., 1999). Recently, a nuclear factor with homology to a heat shock factor (HSF; ten Hove et al., 2010), which encodes the *SCHIZORIZA* gene (Mylona et al., 2002), was also linked to radial patterning. Despite the potential for heat shock protein (HSP) or HSFs to play roles in root hair development and patterning, due to the obvious exposure to changing environmental conditions, genetic evidence for key genes remains poorly characterized.

A large number of genes encode the HSPs and HSFs. HSPs were named as heat response proteins (Ritossa, 1962; Tissiers et al., 1974), but since have been linked to a multitude of biological processes including microtubules stabilization, anti-apoptosis, refolding of a protein in non-native status, regulation of steroid hormone receptors (Kregel, 2002), protein translocation, protein folding reviewed by (Al-Whaibi, 2011), cell proliferation (Pechan, 1991) and at least with regard to fungi, HSPs are involved in signaling (Panaretou and Zhai, 2008). Among the HSPs the most studied are the HSP70 (Bakau and Horwich, 1998) and the DnaJ (Nakamoto and Vígh, 2007; Siddique et al., 2008). DnaJ is a member of the Hsp40 family of molecular chaperones, which is also known as J-protein family (Walsh et al., 2004). In *Arabidopsis*, the J-family encompass as many as 120 members classified in four sub-types (Rajan and D’Silva, 2009). J-protein type I, share all motifs found in a DnaJ a highly conserved N-terminal J-domain, followed by a glycine/phenylalanine rich region, a zinc-binding cysteine rich region and a variable C-terminal region. The remaining types display a simplified structure, with type II, missing the Zinc finger domain and type III displaying only the J-domain. Type IV, are J-like protein with a large similarity to the J-domain but missing the recognition motif HPD (Siddique et al., 2008; Rajan and D’Silva, 2009). DNAJ/ J-domain proteins are best know as co-chaperones working in client binary complex association with HSP70 (Minami et al., 1996; Miernyk, 2001; Qiu et al., 2006; Summers et al., 2009; Jelenska et al., 2010; Bekh-Ochir et al., 2013).

Herein, during a screen of T-DNA *A. thaliana* mutants for defective root hairs we identified that mutations in a type III J-protein (AtDjC17) gene and we aimed to study the transcriptional and genetic features that contribute to root development.

## MATERIALS AND METHODS

### PLANT MATERIAL AND GROWTH CONDITIONS

*Arabidopsis thaliana* (L.) Heynh (*Arabidopsis*) ecotype Colombia-0 was used in all experiments. The T-DNA insertional alleles [At5g23240, germplasm SALK_008678 (*Atdjc17-1-1*) and SALK_024726C (*Atdjc17-1-2*)] were obtained from the *Arabidopsis* Biological Resource Center (ABRC, Ohio State University). Seeds were surface sterilized and vernalized at 4^∘^C for 2 days in the darkness prior to plating them on strength Murashige and Skoog (MS) basal salts medium (pH 5.7; Duchefa, Holland) solidified with 0.8% agar. Seeds were germinated under 16 h light; 8 h darkness conditions at a constant temperature of 22^∘^C. Seeds were plated as above described and plates were vertically positioned and incubated in dark grown (22^∘^C) condition. The phenotypes of *Atdjc17-1-1* and *Atdjc17-1-2* were compared to that of wild-type (WT) during plant growth and development. Plants were grown in MetroMix 360 (Sun Gro Horticulture) in a temperature controlled environmental chamber (22^∘^C; Adaptis, Conviron).

### GENOTYPING OF THE MUTANT LINES

Homozygosity of the knockout lines *Atdjc17-1-1/Atdjc17-1-2* was verified by polymerase chain reaction (PCR)-based genotyping, primers sequences are given in supporting information Table S1. Total plant DNA was extracted as previously described (Rogers and Bendich, 1985). For PCR purposes the DNA concentration was standardized to 100 ng μl^-1^ in Tris pH 8.0 (10 mM).

### MICROSCOPY

Imaging and quantitation of seedling phenotype employed fluorescence stereomicroscopy (Olympus MVX) and ImageJ (National Institutes of Health, Bethesda, MD, USA). Statistical analysis comparing *Atdjc17* and WT plants used PRISM4 (Graphpad, La Jolla, CA, USA) and Minitab (Minitab Inc., USA). Seedling phenotypes, including root hair and epidermal patterning defects were examined consistently at 7-d post-germination. Seedlings were grown vertically in strength MS agar. Root hair length measurements were averaged across the entire root. To examine the pattern of epidermal development in a uniform spatial region of the root we documented cell area in the region covering 0.65 mm of root, initiating approximately 2 mm above the root cap. Average cell length and area determinations for each trichoblast/atrichoblast cell used area measurement output after tracing the polygon via the freehand selection tool (ImageJ) and pixel-number^2^ converted to μm^2^. Due to the 3-dimensional nature of the root structure only those root hairs visible in the optical plane were counted. Transverse root sections were made as described (Hung et al., 1998) whereby roots were embedded in 3% molten agarose and hand sectioned using double-edged razor blade. The sections were stained with calcofluor-white (Sigma, USA) stain and visualized under fluorescence stereomicroscope (Olympus MVX; DAPI filter). For β-glucuronidase (GUS) histochemical assay, staining solution was prepared according to (Guivarc’h et al., 1996). The seedlings were cleared, sectioned as above and counter stained with 0.05% Ruthenium red according to (Hassan et al., 2010) before visualization. Propidium iodide staining was performed as described in (Nawy et al., 2005). Accordingly 7-d post-germination seedlings were stained with 10 mgL^-1^ propidium iodide for 30 s to 2 min, rinsed and mounted on water. Microscopy was performed on an Olympus FV1000 laser scanning confocal microscope using a 63 × N.A 1.4 water-immersion objective. The microscope is equipped with lasers for excitation wavelengths ranging from 405 to 633 nm and propidium iodide stain was excited using the DsRed setting in the Olympus Fluoview software (Olympus). All image processing was performed by using Olympus Fluoview software (Olympus) and ImageJ (W. Rasband, National Institutes of Health, Bethesda, MD, USA) software.

### CONSTRUCTION OF REPORTER AND OVEREXPRESSION LINES. SELECTION AND EXPRESSION ANALYSIS OF TRANSGENIC LINES

The *AtDjC17* transcript accumulation was assayed by fusing the *AtDjC17* promoter to the GUS (Jefferson et al., 1987) reporter gene through a promoter::*uidA* fusion construct. A 1.5 Kb putative promoter region was PCR-amplified with the specific primers ATDJC17P-F/ATDJC17P-R (Supporting information Table S1) and the PCR amplified product was cloned into pCXPGUS ZeBaTA vectors (Chen et al., 2009). For overexpression studies, the open reading frame was PCR amplified from genomic DNA using primers ATDJC17G-F/ATDJC17G-R and the amplicon (1.45 Kb) was cloned into the pCXSN vector (Chen et al., 2009) under the constitutive expression of the Cauliflower mosaic virus (CaMV)-35S promoter (35S). Sequence verified clones were transformed by electroporation into *Agrobacterium tumefaciens* hypervirulent strain GVS3101. *Arabidopsis* plants were transformed (Clough and Bent, 1998) and homozygous alleles selected using the selectable marker hygromycin (25 μg/ml, Duchefa). Homozygous T3 plants from independent transformants were used in subsequent studies. T-DNA lines were complemented by restoring *AtDjC17* under the control of the native promoter. The native promoter was PCR amplified and cloned within *KpnI* and *HindIII* sites of the pMDC32 vector replacing the 2X35S promoter. The full length *AtDjC17* cDNA was cloned within the *AscI* and *PacI* sites completing the fusion cassette. For complementation, T-DNA lines were floral dipped and selected for hygromcyn resistance. T2/T3 generations were used for phenothypical characterization.

### GENE EXPRESSION STUDIES

Sterilized *Atdjc17* and WT seed were germinated and grown vertically on strength MS agar plates in 16:8 light:dark conditions for 7-d. Root was rapidly excised from batches of approximately 200 seedlings using a surgical blade in aseptic conditions and snap frozen in liquid nitrogen. Total RNA was extracted using QIAGEN RNAeasy Plant mini kit and treated with DNAse I (Fermentas, LifeSciences) according to the manufacturer’s instructions. Up to 2 μg of the extracted total RNA was used for single stranded cDNA synthesis using High capacity cDNA reverse transcription kit (Applied Biosystems). The final volume was diluted fourfold and 2 μl of the synthesized cDNA (100 ng) was used in the subsequent RT-PCR reactions. Quantitative real time PCR was conducted using Fast SYBR®; Green Mastermix (Applied Biosystems) or HOTFIREPOL®; EvAGreen®; mastermix (OAK Biotechnologies LLC, USA) with StepOne^TM^ Real-Time PCR system (Applied Biosystems). For the RT-PCR reaction the following conditions were used: 1 cycle of initial denaturation at 95^∘^C for 10 or 15 min accordingly to the master mix employed, followed by 40 cycles of denaturation at 95^∘^C for 15 s and annealing/extension at 60^∘^C for 30 s; followed by melting curve analysis. Actin 2 was used as internal control (Supporting information Table S1), with three-pooled biological replicates and three technical replicates. Primers for RT-PCR where possible were taken from referenced sources or designed using PRIMER3 (http://www.embnet.sk/cgi-bin/primer3_www.cgi; Supporting information Table S1).

## RESULTS

### MUTATIONS IN *Atdjc17* CAUSED ALTERED ORGANIZATION OF ROOT HAIR POSITION IN ATRICHOBLAST VERSUS TRICHOBLAST CELL FILES

In a screen for altered root hair (H-cell) occurrence, we identified a causal mutation in *Atdjc17-1-1* (At5g23240). Motif analysis showed that *AtDjC17* contained a J-domain motif and was broadly classified as a J-protein type III (Rajan and D’Silva, 2009). To further confirm the root hair phenotype, two alleles were isolated and correspond to *Atdjc17-1-1* and *Atdjc17-1-2.* Homozygosity for the insertion of a T-DNA into the *AtDjC17* exon was verified by PCR (see Supplementary material Figure S1 for insertion position). Further, *AtDjC17* mRNA abundance was examined in WT as well as *Atdjc17-1-1* and *Atdjc17-1-2* plants by qRT-PCR and these data revealed no detectable *AtDjC17* mRNA for either allele. Thus, we concluded that both *Atdjc17-1-1* and *Atdjc17-1-2* were null alleles.

**FIGURE 1:**
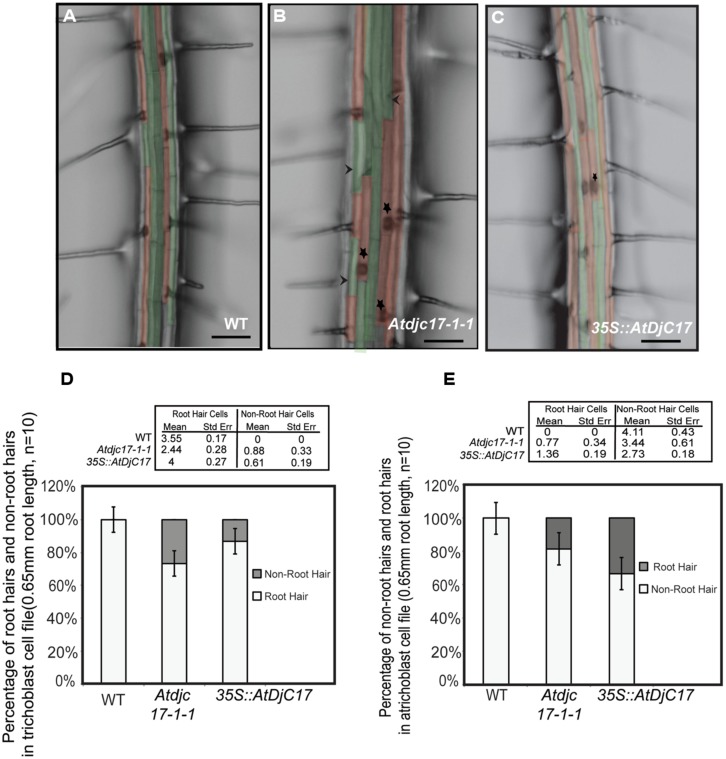
**Trichoblast and atrichoblast cells are arranged randomly in *Atdjc17* and *overexpressor* mutants. (A–C)** False colored stereomicroscope images of wild-type (WT; **A**), *Atdjc17-1-1* **(B)** and *35S::AtDjC17* **(C)** illustrate the patterning of root hair cells (red) and non-root hair cells (green) in cell files. Stars represent ectopic root hairs in an otherwise atrichoblast cell file. Arrowheads represent the presence of non-root hair in an otherwise trichoblast cell file in *Atdjc17-1-1*. **(D,E)** Root hair and non-root hair cells in trichoblast and atrichoblast cell files were counted in WT, *Atdjc17-1-1* and overexpressor *35S::AtDjC17.*

**FIGURE 2:**
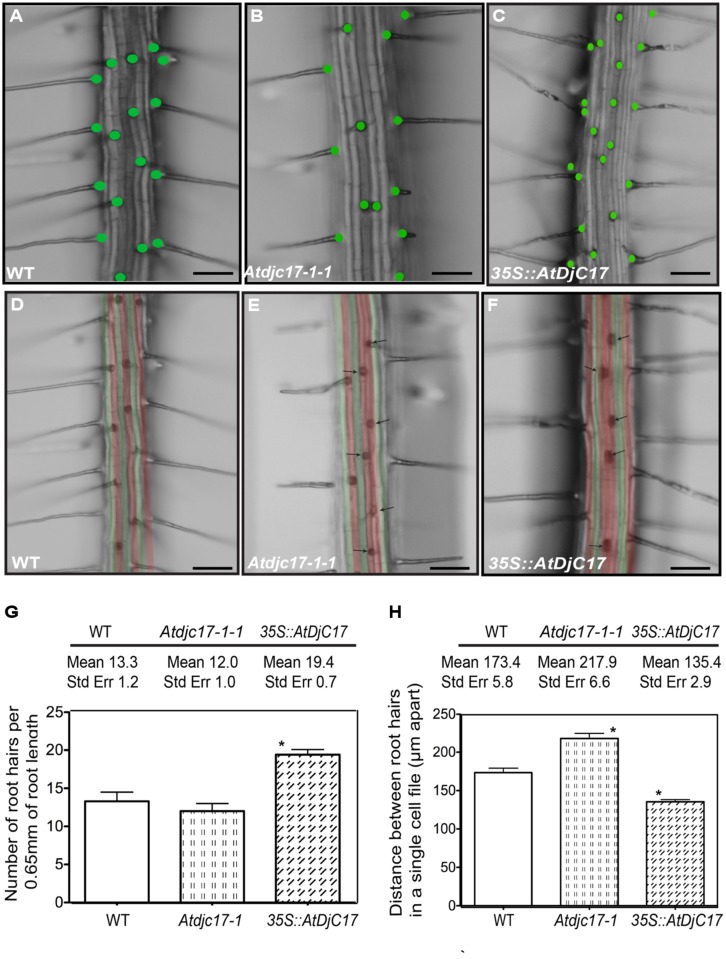
**Mutations and overexpression in *ATDJC17* cause root hair alteration and ectopic root hair production. (A–C)** Variation in number of root hairs is illustrated in *Atdjc17-1-1* and *35S::AtDjC17* mutants as compared to WT plants. Green dots highlight the presence of root hair at the site. **(G)** Average number of root hairs determined from a total of 10 roots. An area approximately 2 mm from the root cap was chosen for the comparison covering 0.65 mm root length. **(D–F)** Stereomicroscope images of WT **(D)**, *Atdjc17-1-1* **(E)** and *35S*::*AtDjC17* **(F)** roots false colored to show the trichoblast (red) and atrichoblast (green) cell files. Arrows in *Atdjc17-1-1* and *35S::AtDjC17* mutant highlights the presence of two trichoblast cell files adjacent to each other illustrating the presence of ectopic root hair. **(H)** Comparison of distance between adjacent root hairs in a single vertical trichoblast cell file in WT **(D)** *Atdjc17-1-1* **(E)** and overexpressor *35S::AtDjC17* **(F)**. *indicates significant difference (*P* ≤ 0.05).

H-cells appeared in adjacent cell files rather than in alternating cell files in both mutant alleles (*Atdjc17-1-1* and *Atdjc17-1-2* respectively, **Figure 1** and supplementary material Figure S2) described as irregular root H emergence. A 31% reduction in H-cells was observed in a trichoblast cell file in *Atdjc17-1-1* (**Figures 1B–D**). As expected, 100% of H-cells were identified in the trichoblast cell file in WT roots (*n* = 10 seedlings; **Figures 1A–D**). In *Atdjc17-1-1* we observed H-cells in atrichoblast cell file (approximately 19.5% H cells), whereas no H formation was observed in atrichoblast cell files in WT. Alongside these data we also observed a quantitative increase in the distance between adjacent H-cells in a cell file in *Atdjc17* alleles compared with WT (217.9 ± 6.6 μm for *Atdjc17-1-1* and 173.4 ± 5.8 μm for WT, *P* < 0.05; **Figure 2H**). Accounting for this phenotype was an observed increase in trichoblast cell area for *Atdjc17* mutants (*Atdjc17-1-1:* 2916 ± 161.6 μm^2^; WT: 2384 ± 104.2 μm^2^, *P* > 0.05), indicative of an expansion defect.

To query whether increasing the transcript abundance for *Atdjc17* would influence root epidermal patterning phenotype, we expressed a *35S::AtDjC17* in WT plants. This resulted in a transgenic plant with 3.5-fold increase in *AtDjC17* transcript in the roots. Visual examination of the *35S::AtDjC17* roots also revealed irregular H-cell occurrence compared with WT. In an opposite fashion than was observed in *Atdjc17* mutants, the *35S::AtDjC17* displayed a 12.5% reduction in H-cells in a trichoblast cell file (**Figures 1C–D**) as compared to 31% in *Atdjc17-1*. Average occurrence of H-cells in the atrichoblast file was 34% (**Figure 1E**) compared to the 19.5% in *Atdjc17.* Additionally, *35S::AtDjC17* displayed a significant increase in the number of H-cells calculated per 0.65 mm of root length i.e., 19.4 ± 0.7 (**Figure 2G**, *P* < 0.05) when compared with the values determined for the WT (**Figures 2A–G**) and *Atdjc17-1-1* (**Figures 2B–G**). This increase was also paralleled by a reduction of the distance between root H-cells (135 ± 2.9 μm, *P* < 0.05; **Figure 2H**) as compared to WT and to the *Atdjc17* mutants. These data support the requirement for AtDjC17 in determining the correct positional distribution of H-cells among epidermis cells in the *Arabidopsis* root.

To further confirm that observed phenotypes in *Atdjc17* were in response to dysfunction in *AtDjC17* we complemented the T-DNA mutant with the *AtDjC17* driven by its native promoter (2000 bp upstream of initiation codon). The resulting complementation line was not discernible from the WT with respect to H-cell frequency and position (Supplementary Figure 3). These data were consistent with the observed phenotypes linked to *AtDjC17*.

### VISUAL EXAMINATION OF *proAtDjC17::GUS* TRANSCRIPT REVEALED LOCALIZATION TO THE STELE CELLS

**FIGURE 3:**
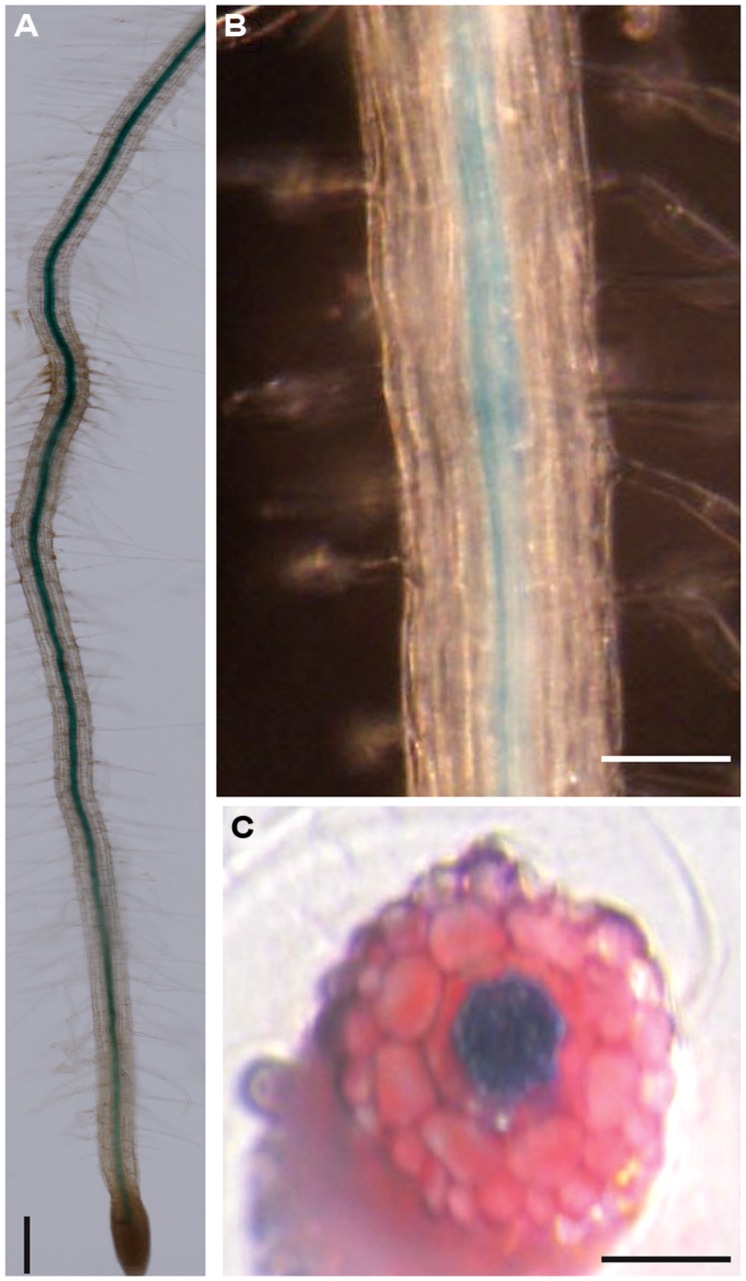
**GUS expression studies. (A,B)** Stele-localized accumulation of *proAtDjC17::GUS* transcript in a 7-d old root. **(A)** Scale Bar 100 μm.**(B)** Scale Bar 50 μm. **(C)** Agarose embedded hand section of pro*AtDjC17::GUS* expressing 7-d old roots displaying a clear stele accumulation of GUS, Scale Bar 50 μm.

To visually determine where the *AtDjC17* transcript was expressed during root development we generated a β-glucuronidase (GUS) reporter fused to the *AtDjC17* promoter. Microscopic examination of 7-day old seedlings indicated a spatially discreet zone of transcript coincident with the stele and no visible abundance in cortical, endodermal or epidermal cell files (**Figures 3A,B**). Cross referencing of the stele expression for *proAtDjC17::GUS* with the cell sorting approach taken by Brady et al. (2007) was not conclusive as *AtDjC17* was expressed found to be expressed in low levels in the stele tissue. Transverse sections of stained and cleared seedlings confirmed that transcript was localized principally in the stele (**Figure 3C**). To also explore whether or not *AtDjC17* transcript abundance was stress dependent or independent in a cell type specific manner, we imposed various stress regimes on the transgenic plants expressing *proAtDjC17::GUS.* These results revealed no shift in expression under stress.

### TRANSCRIPT ANALYSIS REVEALS DIFFERENTIAL EXPRESSION IN ROOT PATTERNING GENES IN *Atdjc17-1* and *35S::AtDjC17* ROOTS VERSUS WT

**FIGURE 4:**
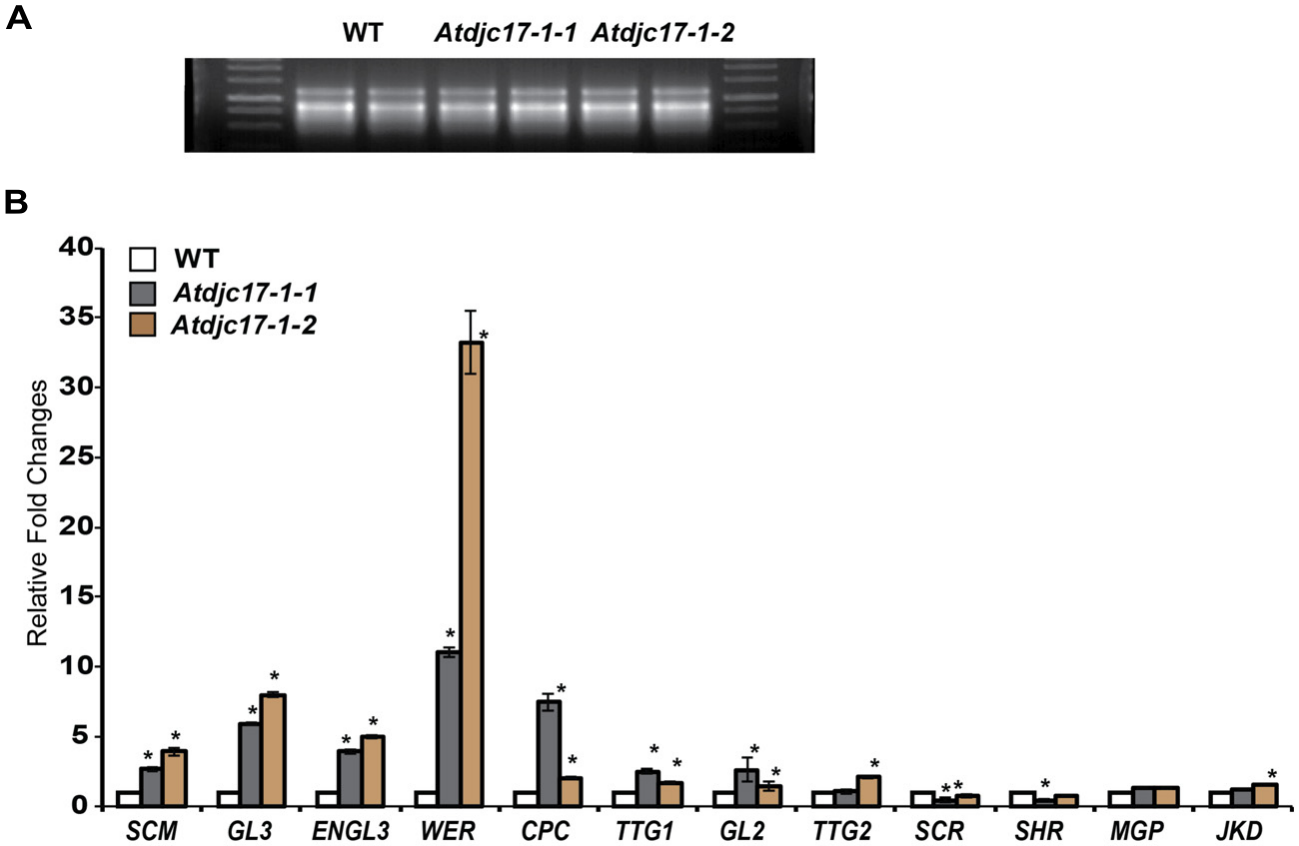
**Gene expression analysis for previously identified regulatory elements of root development. (A)** RNA equal loading of WT and *Atdjc17* mutant lines (*Atdjc17-1-1, Atdjc17-1-2*). **(B)** Relative fold changes determined on whole root sample for *SCRAMBLE* (*SCM*); *GLABRA3* (*GL3*); *Enhancer of GLABRA3* (*ENGL3*);*WEREWOLF* (*WER*); *CAPLICE* (*CPC*); *TRANSPARENT TESTA 1* (*TTG1*);*GLABRA2* (*GL2*); *TRANSPARENT TESTA 2* (*TTG2*); *SCARECROW* (*SCR*); *SHORTROOT* (*SHR*); *MAGPIE* (*MGP*) and *JAKDOW* (*JKD*). Error bars indicate standard deviation. *indicates significant difference (*P* ≤ 0.05).

**FIGURE 5:**
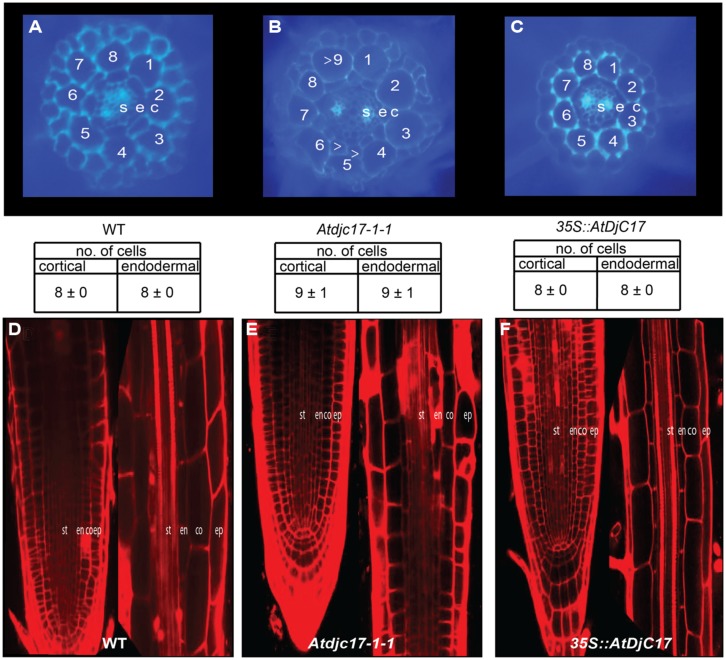
**Examination of cell division in WT, *Atdjc171-1* and *35S::AtDjC17* roots: agarose embedded hand sections stained with calcofluor-white stained and confocal microscopic images of propidium iodide stained WT, *Atdjc17-1-1* and *35S::AtDjC17* mutant roots. (A)** WT section. **(B)** *Atdjc17-1-1* section showing additional cell division in the cortical and endodermal layers. Note that the divisional pattern seems anticlinal in nature. Arrow heads indicate ectopic divisions. **(C)** 35S::*AtDjC17* section with no evidence of altered cortical and endodermal cell numbers. **(D)** 7-day old post-germination WT root tip and zone above the meristematic tip in WT root. **(E)** *Atdjc17-1-1* root tip with associated zone above the meristematic tip.**(F)** *35S::AtDjC17* root tip and elongation zone. st, stele; en, endodermis; co, cortex; ep, epidermis.

The expression levels of known epidermal and radial patterning genes was explored. Here, we examined the LRR-RLK (*SCM),* the bHLH transcription factors *GLABRA3* (*GL3*) and *EGL3,* the R2R3-MYB transcription factor *(WER),* the small single-repeat R3-MYB transcription factor *CAPRICE (CPC),* the WD-repeat *TTG1,* the WRKY transcription factor *TRANSPARENT TESTA GLABRA2 (TTG2),* the homeodomain-leucine-zipper transcription factor *GLABRA* 2 (*GL2),* the basic-leucine zipper transcription factor *SCARECROW (SCR)*, the transcription factor *SHR*, and zinc finger proteins *JACKDAW* (*JKD*) and *MAPGPIE* (*MGP*; Galway et al., 1994; Di Laurenzio et al., 1996; Masucci et al., 1996; Wada et al., 1997, 2002; Lee and Schiefelbein, 1999; Helariutta et al., 2000; Schellmann et al., 2002; Sabatini et al., 2003; Zhang et al., 2003; Bernhardt et al., 2005; Koshino-Kimura et al., 2005; Ishida et al., 2007, 2008; Kwak and Schiefelbein, 2007; Welch et al., 2007; Hassan et al., 2010) in *Atdjc17* alleles compared with WT. *TTG2* amongst the epidermal patterning regulators and *JKD* and *MGP* involved in radial and epidermal patterning were not differentially expressed when compared to WT (**Figure 4**). Results showed a significant (*P* ≤ 0.05) up-regulation of *GL3, SCM*, *EGL3, WER, CPC,* and *TTG1* as compared to WT (**Figure 4**). The principle exceptions were *SCR* and *SHR*, which were down-regulated (*P* < 0.05, **Figure 4**). This trichoblast and atrichoblast specific transcripts increased abundance is consistent with both irregular H-cell development and reduced frequency of H-cells. *SCR* transcript was down-regulated in the *Atdjc17* mutant root (**Figure 4**) moreover *SCR* has been shown to cause a loss and coupling of endodermal and cortical cell layers (Di Laurenzio et al., 1996). However, mutants with loss in the *AtDjC17* gene product did not exert as dramatic effects as other characterized mutants such as *shr*, as evidenced by agarose embedded hand-sections or propidium iodide staining (**Figure 5**). This down-regulation of *SCR* transcript likely resulted from an up or downstream regulation, such as *SHR*, which was also down-regulated in *Atdjc17*. Nevertheless, a cell division increase was quantified in cortical (Increased cell division: *Atdjc171-1/1-2*: 9 cells/section ± 1 vs. WT 8 cells/section ± 0, *n* = 15) and endodermal layers (Increase in cell frequency in endodermal layer *Atdjc171-1/1-2*: 9 cells/section ± 1 vs. WT 8 cells/section ± 0, *n* = 15) of the *Atdjc17* mutant, which was consistent with a “scrambling” of expression among transcripts involved in epidermal patterning.

**FIGURE 6:**
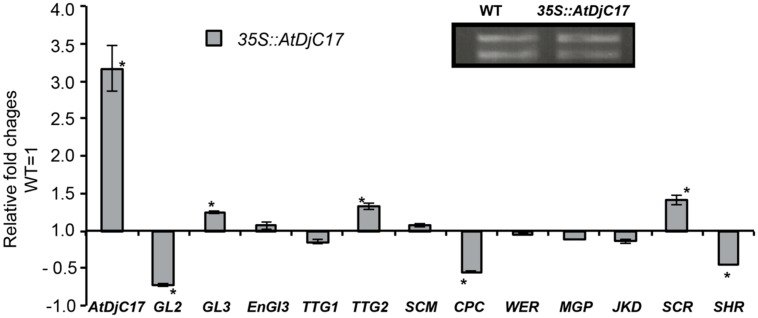
**Root expression analysis of the overexpressor of *AtDjC17.*** Relative fold changes determined on whole root sample for 35S::AtDjC17 and 12 main regulators of root development. Error bars indicate standard deviation. *indicates significant difference (*P* ≤ 0.05).

Transcriptional analyses of *AtDjC17* and 12 known regulators of root development (**Figure 6**) were investigated in the overexpressor of *35S::AtDjC17*. As expected, *35S::AtDjC17* increased *AtDjC17* transcript levels but it was also noted a down-regulation of *GL2,* which was consistent with the ectopic root hair phenotype previously described (**Figure 1**). In addition to *35S::AtDjC17* also the transcript levels of *GL3*, *TTG2*, and *SCR* were also up-regulated. Consistently with *GL2* also *CPC* was found down-regulated along with *SHR*, whereas the remaining regulators were found to be unchanged.

### CORTICAL AND ENDODERMAL CELL LAYERS DISPLAY ABERRANT DIVISIONS IN *Atdjc17* BUT NOT IN *35S::AtDjC17*

Phenotypes associated with the dysfunction in the stele expressed *SHR*, and ground tissue stem cell expressing *MGP* and *JKD* genes include irregular pattern formation in the cortical and endodermal cell layers (Helariutta et al., 2000; Welch et al., 2007; Koizumi et al., 2011; Ogasawara et al., 2011). Although *shr* has a far more severe effect on plant growth and development than what we have documented for *Atdjc17* alleles (**Figures 1 and 2**; Supplementary material Figure S2) *Atdjc17* subtle phenotypes are more similar to the one documented for *mgp* and *jdw.* Using laser scanning confocal microscopy, we examined the longitudinal cell file development of *Atdjc17* and WT using propidium iodide to fluorescently label the cell walls. Here, the cortical and endodermal cell layers in *Atdjc17* root did not show obvious changes in cell division relative to WT (**Figures 5D–F**). By contrast, when we examined cross sections of WT, *Atdjc17* and *35S::AtDjC17* seedling roots it was evident that the cortical (co) and endodermal (en) layer displayed increased frequency of cell divisions in the *Atdjc17* mutants (co: 9 cells per section ± 1, *n* = 15; en: 9 cells per section ± 1, *n* = 15) compared to the WT (8 cells per section ± 0, *n* = 15; **Figures 5A–B**). On the contrary, *35S::AtDjC17* did not show observable alteration in divisions in either cortical cells or endodermal cell layers (**Figure 5C**). Absence of visible alteration in the longitudinal propidium iodide stained section suggests an anticlinal division alteration only identifiable through cross sections. Changes in the number of cells in the cortical layer directly influences the frequency of trichoblast cells in the epidermal layer due to the position requirement for contact with two underlying cortical cells (Kwak and Schiefelbein, 2007), which was consistent with the observed aberrant H-cell occurrence visualized in *Atdjc17* mutants (**Figure 1**). The modest changes in root patterning (increase cell division: *Atdjc171-1/1-2*: 9 cells/section ± 1 vs. WT 8 cells/section ± 0, *n* = 15) and development (increase in cell frequency in endodermal layer *Atdjc171-1/1-2*: 9 cells/section ± 1 vs. WT 8 cells/section ± 0, *n* = 15) displayed similarity to *mgp* and *jkd* (Welch et al., 2007; Ogasawara et al., 2011).

## DISCUSSION

Mutations in *AtDjC17* identified in this study, caused aberrant cell fate determination and cell divisions in ground tissue layers in *Arabidopsis* roots. Existing literature supports DNAJ/J-domain family proteins functioning as co-chaperones working in association with HSP70 class proteins (Miernyk, 2001; Qiu et al., 2006; Summers et al., 2009; Jelenska et al., 2010; Bekh-Ochir et al., 2013) and based on the observed root phenotypes, we hypothesized that *AtDjC17* would plausibly be required for cell fate determination by acting in a client:binary complex with a cognate HSP70 as a protein chaperone to fortify key stage(s) in the pathway. We envisioned that chaperone function could be important, particularly at the epidermis, where exposure to the adjacent soil environment may require such a chaperone due to environmental stress. However, an unexpected feature of the *AtDjC17* was its prominent expression in restricted to stele tissue of the *Arabidopsis* root (**Figure 3**). This is despite H-cell irregularity phenotypes being observed in the epidermis (**Figure 1**). Interestingly, dysfunction in genes encoding the zinc finger proteins *JKD* and *MGP* resulted in a similar syndrome of defective epidermal cell fate determination and division defects despite transcription being localized outside of epidermal tissue. *JKD* expression is localized in ground tissue, quiescent center (QC) and to a lesser extent in mature cortical cells and is known to limit *SHR* and control cell divisions that give rise to endodermal and cortical layers (Welch et al., 2007; Hassan et al., 2010). *SHR,* a GRAS family transcription factor is expressed in stele tissue (Welch et al., 2007) and influences root development by influencing asymmetric divisions that give rise to ground tissue as well as endodermal cell identity. It does this in part by regulating *SCR,* another GRAS family transcription factor (Helariutta et al., 2000). Both *SHR* and *SCR* affect overall root development as mutations in these genes causes supernumerary and replacement of cortical/endodermal cell layer with a single ground cell layer having heterogeneous cell identity. Therefore, matching expression for *AtDjC17* and *SHR* suggests possible functional influence in the same pathway. We propose a model whereby a chaperone complex involving *AtDjC17* could be influencing this pathway in a non-cell autonomous fashion that involves *SHR*, *SCR, JKD,* and *MGP*.

Indeed, examining gene expression of SHR and SCR in *Atdjc17* supports these data. Quantitative real time (Q-RT) PCR revealed down-regulation of *SCR.* We anticipated that down-regulation of *SCR* in *Atdjc17* would be mirrored by *SHR*, which was confirmed by Q-RT PCR (**Figure 4**). By contrast, in the whole root samples from *Atdjc17*, trichoblast, and atrichoblast specific expressed transcripts [atrichoblast (*WER, GL2, CPC*); trichoblast (*EGL3, GL3,*)] both displayed increased abundance relative to WT. Considering the mixed identity among epidermal cell files observed as H-cell-fate irregularities, the observed increased transcript abundance among these cell type specific transcripts was not unexpected, but does not conclusively suggest any single element as responsive. Alternatively, Q-RT PCR on whole root samples might not be a sensitive method to uncover differences in cell type specific transcript levels. Cell type specific Q-RT PCR on *Atdjc17* may provide better understanding of the differences in gene expression for epidermal patterning specific genes.

Intriguingly, expression levels of *JKD* and *MGP,* were not differentially expressed in *Atdjc17* further suggests that *AtDjC17* functions independently of these factors. These data did not support our hypothesis of transcriptional linkage to the zinc finger proteins *JKD* and *MGP* but did identify transcriptional association to *SCR/SHR*. Given its critical requirement for root development, it is indeed plausible that molecular chaperone function for *SHR* would be important to safeguard the root developmental program. Taken together, *JKD* null mutations cause ectopic periclinal divisions in the cortical and endodermal layer but also an ectopic root hair development in a non-cell autonomous fashion (Welch et al., 2007; Hassan et al., 2010). Hence, while the localization of *AtDjC17* expression to stele cells partially overlapped with *SHR* the phenotype of *Atdjc17* more closely resembled *jkd,* although with a far less severe impact on cell division and whole plant morphogenic phenotypes. The position dependence needed to acquire H-cell versus N-cell fate in epidermal cells (Dolan, 2006) is such that ectopic divisions within the cortical cells observed in transverse cross sections of the *Atdjc17* root could explain the corresponding irregular pattern of H-cell emergence (**Figure 1**). Further studies are needed to assign biochemical association between possible targets of the *AtDjC17* co-chaperone, in addition to isolation of the anticipated cognate HSP70. Although many genes have been identified in root development (Guimil and Dunand, 2006) no prior evidence supports a requirement for a DNAJ-HSP40 for epidermal cell fate determination, and results from *Atdjc17* raise the intriguing possibility of a HSP complex playing a chaperone role in root development.

## AUTHOR CONTRIBUTIONS

Carloalberto Petti carried out the phenotyping and genotyping of the T-DNA, the RT analyses and the transgenesis and drafted the manuscript. Meera Nair helped with the phenotyping and genotyping of the T-DNA lines and drafted the manuscript. Seth DeBolt conceived the study and drafted the manuscript. All authors read and approved the final manuscript.

## Conflict of Interest Statement

The authors declare that the research was conducted in the absence of any commercial or financial relationships that could be construed as a potential conflict of interest.

## SUPPLEMENTARY MATERIAL

The manuscript contains a supplementary table (Table S1) listing the primers employed for genotyping, cloning, and for RT-PCR studies. Additionally it also contains three supplementary figures displaying the position of the insertion (Figure S1); detailed characterization of root hair phenotype of *Atdjc17-1-2* (Figure S2); complemented T-DNA lines by native promoter driven construct (Figure S3). The Supplementary Material for this article can be found online at: http://www.frontiersin.org/journal/10.3389/fpls.2014.00532/abstract
