# Supplementary material for: The involvement of J-protein AtDjC17 in root development in Arabidopsis
Source: Front Plant Sci. 2014 Oct 8;5:532. doi: 10.3389/fpls.2014.00532 (PMC4189540; doi:10.3389/fpls.2014.00532)
Supplement: Supplementary file 1 [file Data_Sheet_1.DOCX]

**The involvement of J-protein AtDjC17 in root development in Arabidopsis**

Carloalberto Petti, Meera Nair and Seth DeBolt^a^

**Affiliations:**

Department of Horticulture, University of Kentucky, Lexington KY USA

Table S1: List of primers employed for genotyping, cloning and for Real Time-PCR

| **Name** | **Function** | **Sequence** | **Ref.** |
| --- | --- | --- | --- |
| **Genotyping** | | | |
| ***Atdjc17-1-1-LP*** |  | 5’-tattgattccgtcgccaatac-3’ |  |
| ***Atdjc17-1-1-RP*** |  | 5’-aatgatggccatatcatgacc-3’ |  |
| ***Atdjc17-1-2-LP*** |  | 5’-GAGTCTATCGCAATCGACGAG-3’ |  |
| ***Atdjc17-1-2-RP*** |  | 5’-TTACTGGAGTCCATCAAACGC-3’ |  |
| ***LBb1-3*** |  | 5’-ATTTTGCCGATTTCGGAAC-3’ |  |
| **Promoter and Gene Cloning** | | | |
| ***ProAtdjc17-F*** |  | 5’-GGTAGTGGTGATGAGATAGTAG-3’ |  |
| ***ProAtdjc17-R*** |  | 5’-ATTTTCCCCTTCTCTGTTTTGGAA-3’ |  |
| ***Atdjc17-F*** |  | 5’-AGCTGTTCAACACAAGCTAGAAGA-3’ |  |
| ***Atdjc17-R*** |  | 5’-AATGTTCTTTGTTACATTTGCAGGT-3’ |  |
|  | **RT-PCR** |  |  |
| ***SCM-F*** |  | 5’-ACATCGATGCGTTGACAAGA-3’ |  |
| ***SCM-R*** |  | 5’-TATCGGCGGTCTAAATCCTG-3’ |  |
| ***SCR-F*** |  | 5’-CAGTTGATGGAGCCAAATCC-3’ |  |
| ***SCR-R*** |  | 5’-AACTGCCTCTCCTTTCCACA-3’ |  |
| ***WER-F*** |  | 5’-TGTCAAAGCTCATGGCAAAG-3’ |  |
| ***WER-R*** |  | 5’-ATCCTCTTCTTGCTCGGTGA-3’ |  |
| ***CPC-F*** |  | 5’-TTGGCGACAGGTGGGAGTTGAT-3’ |  |
| ***CPC-R*** |  | 5’-AACGACGCCGTGTTTCATAAG-3’ |  |
| ***GL3-F*** |  | 5’-ACATTGGTGAAGGAATGCCTGGAC-3’ | 1 |
| ***GL3-R*** |  | 5’-TTACTATCCGCCGTATGAGCGTTG-3’ | 1 |
| ***EGL3-F*** |  | 5’-TGAAACCGCCGATAGCAAAG-3’ | 1 |
| ***EGL3-R*** |  | 5’-CTCCAAGAAACGGGAAGCAA-3’ | 1 |
| ***TTG1-F*** |  | 5’-GCGATTTCCTCCGTCTTTGG-3’ | 1 |
| ***TTG1-R*** |  | 5’-CGCTCGTTTTGCTGTTGTTG-3’ | 1 |
| ***TTG2-F*** |  | 5’-CCCCACAACTTTCTAAGCAAACA-3’ | 1 |
| ***TTG2-R*** |  | 5’-TGCTTAGGAAGTTGTGAGTGAAG-3’ | 1 |
| ***GL2-F*** |  | 5’-ATGAAGCTCGTCGGCATGAGTGGG-3’ | 1 |
| ***GL2-R*** |  | 5’-TGGATTGCCACTGAGTTGCCTCTG-3’ | 1 |
| ***SRT-F*** |  | 5’-GGTGTTTGGTCGATGGTACA-3’ |  |
| ***SRT-R*** |  | 5’-CTCAAAGCCCATCATCAACC-3’ |  |
| ***MGP-F*** |  | 5’-GGTTCTTTGCTTCGTTTGGA-3’ |  |
| ***MGP-R*** |  | 5’-CCGCATTCCCAATATCAACT-3’ |  |
| ***JKD-F*** |  | 5’-TTGCTCCATTGGGTTGATTA-3’ |  |
| ***JKD-R*** |  | 5’-CAACCTTTGTCCCCACATTC-3’ |  |
| ***ACT2-F*** |  | 5’-GGCTTAAAAAGCTGGGGTTT-3’ |  |
| ***ACT2-R*** |  | 5’-TTGTCACACACAAGTGCATCA-3’ |  |

1-**Ishida T, Hattori S, Sano R, Inoue K, Shirano Y, Hayashi H, Shibata D, Sato S, Kato T, Tabata S, Okada K, Wada T** (2007) Arabidopsis *TRANSPARENT TESTA GLABRA2* Is Directly Regulated by R2R3 MYB Transcription Factors and Is Involved in Regulation of *GLABRA2* Transcription in Epidermal Differentiation. *The Plant Cell Online* **19**: 2531-2543

**Figure Legend**

**Supplementary figure 1. Gene model and position of T-DNA insertions.** Graphical representation of the gene model associated with AtDjC17 with highlighted the T-DNA insertion.

**Supplementary figure 2. Fig. 2. Mutations in *ATDJC17* cause root hair alteration and ectopic root hair production.** Histograms showing frequency of root hair length in WT (A) and *Atdjc17-1-2* (B). Whole roots of 10 seedlings were used. (C-D) Root hair and non-root hair cells in trichoblast and atrichoblast cell files were counted in WT, and *Atdjc17-1-2.* **(E***)* Variation in number of root hairs as determined in *Atdjc17-1-2* and as compared to WT plants. An area approximately 2 mm from the root cap was chosen for the comparison covering 0.65mm root length. **(F)** Comparison of distance between adjacent root hairs in a single vertical trichoblast cell file in WT (A) and *Atdjc17-1-2* (E). Asterisk represents significance based on P<0.05.

**Supplementary figure 3. Phenothypical characterization of complemented T-DNA line.** The T-DNA lines were complemented by the native promoter driven *AtDjC17* overexpression. T3 lines were assed for root hair phenothypical characterization. Stereomicroscope images of WT (A) and *Atdjc17-1-1* (B) which displayed no difference in root hair positioning as compared to WT. (C-D) Histograms showing comparable frequency of root hair length in WT (C) and Pro*Atdjc17 x Atdjc17-1* (D).

**Figure S1**

**
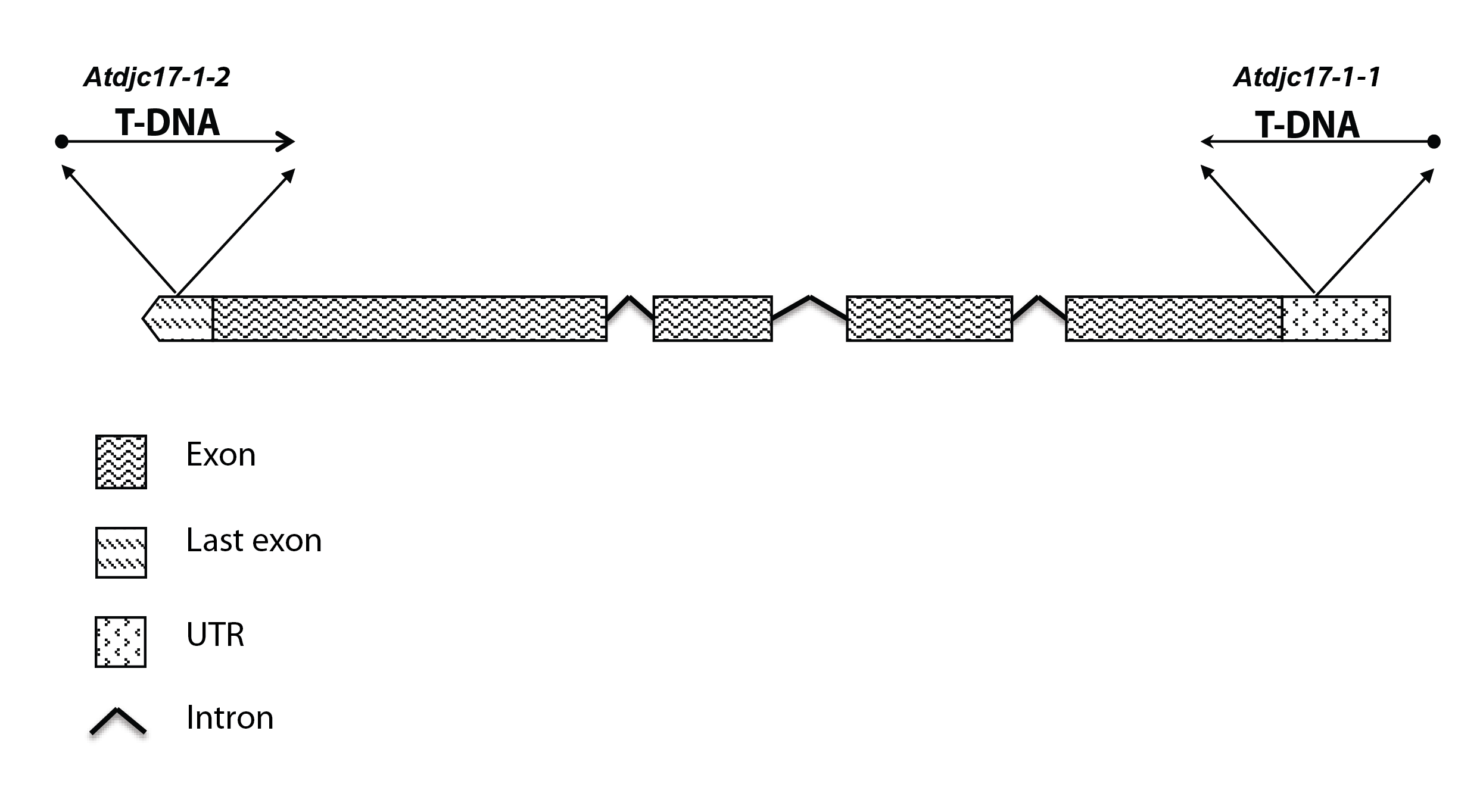
**

**Figure S2**

**
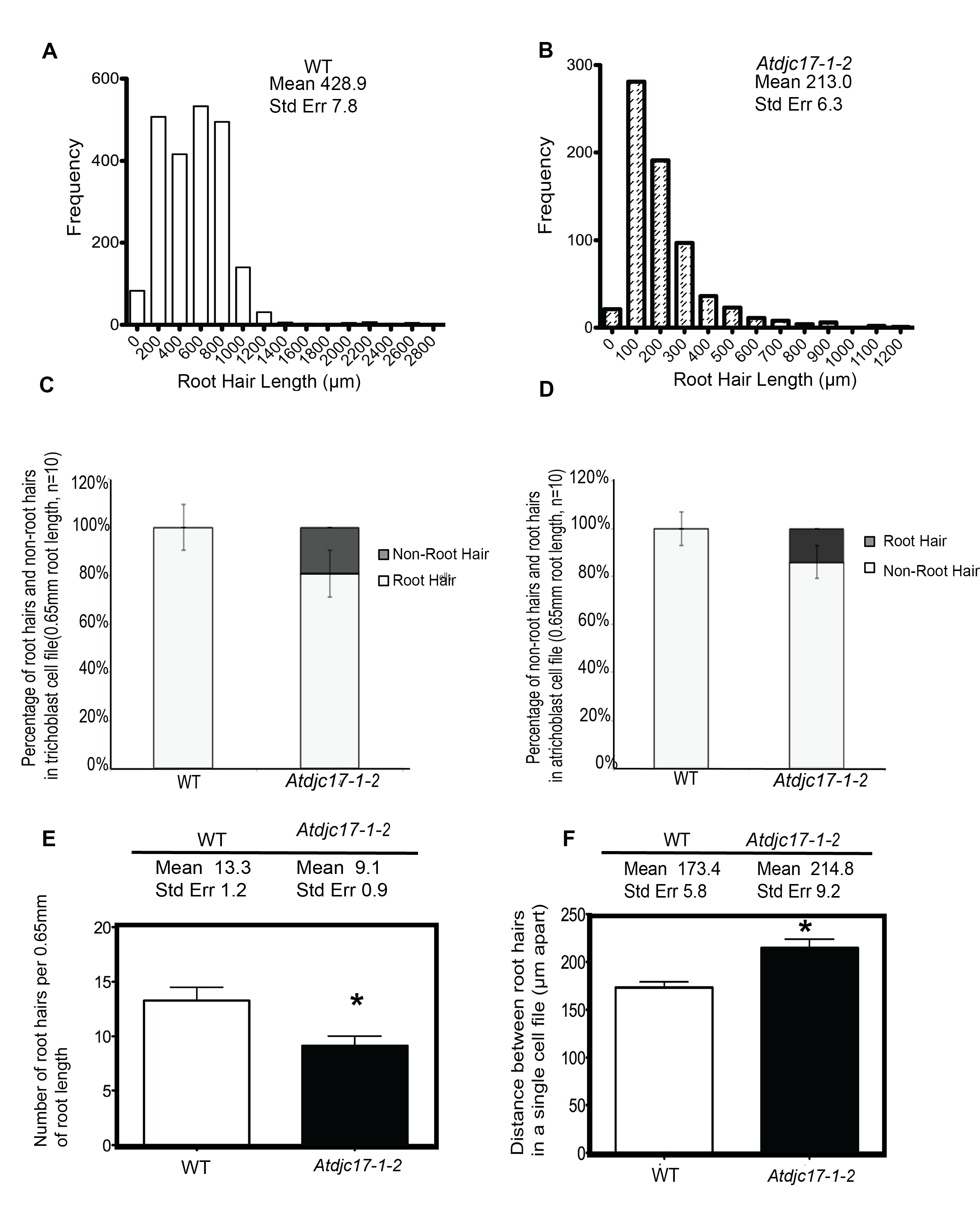
**

**Figure S3**

**
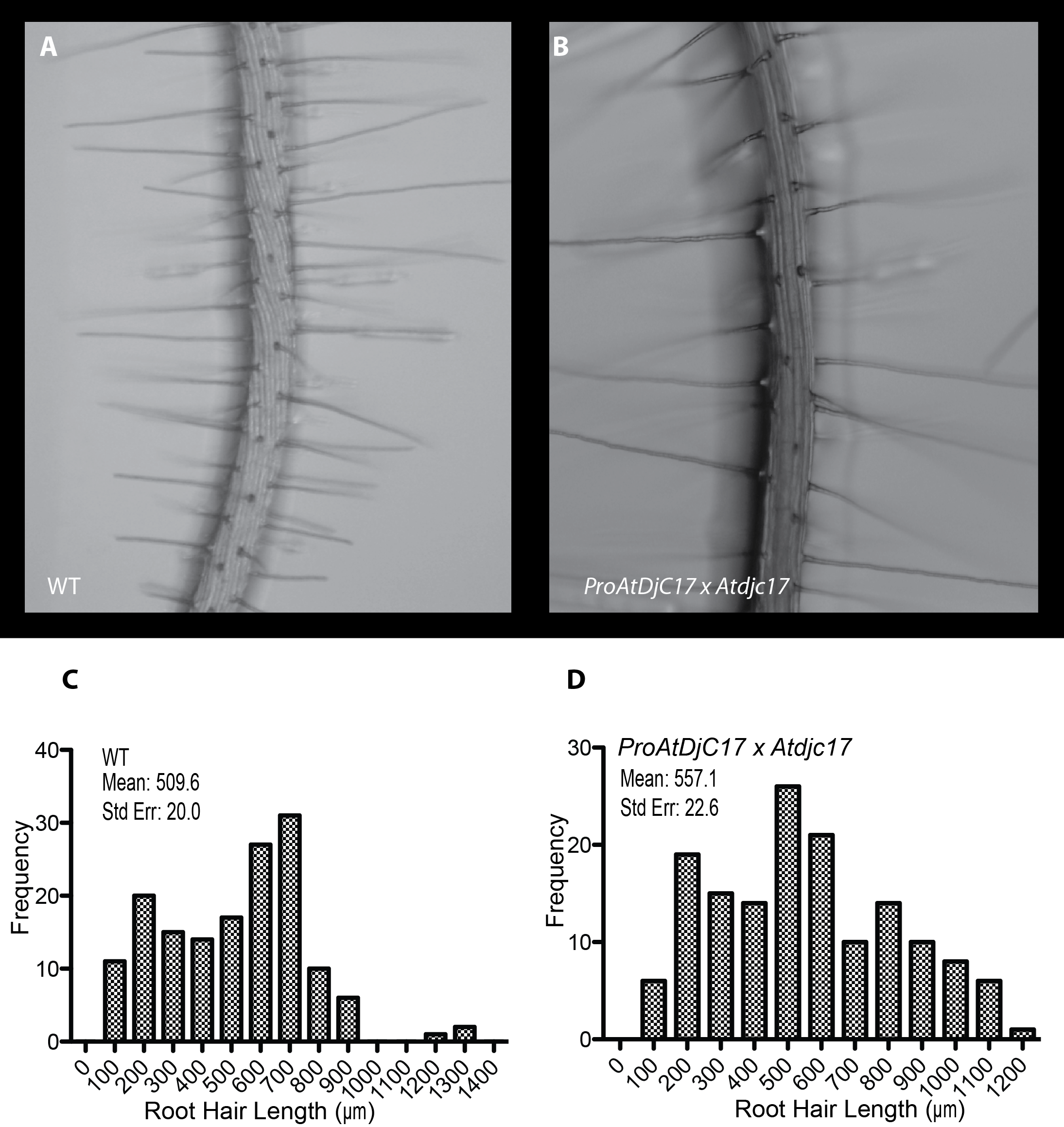
**
